# Foreign Notes and Excerpta

**Published:** 1868-10-15

**Authors:** 


### *FOREIGN NOTES AND EXCERPTA.*

*Influence of Diet upon the Mother's Milk. Translated by D. F. Condie. M.D.*

The contradictory opinions that are entertained in respect to the influence of diet upon the quantity and quality of the milk, induced Dr. Subotin, of Petersburg (*Virchow's Arch.*, vol. xxxvi. p. 561), to institute a series of experiments to settle, as far as possible, the question. His investigations led him to the following conclusions:

1. That animal food increases the daily yield of milk, while a diet of vegetables diminishes it. Food of a fatty nature caused a marked diminution of the milk, and even, when persisted in, its entire suppression.

2. The character of the food had an evident influence upon the relative proportions of the several elements which enter into the composition of the milk. By an animal diet the amount of the solid matters was increased, and this increase was especially shown in an augmentation of fatty material. The increase of casein was less evident. The augmentation of these two substances in the milk was not merely relative but absolute; the daily amount of milk secreted being increased by animal food. The proportion of its albuminous and saline ingredients underwent scarcely any appreciable change. Under the use of an animal diet there was not detected any large reduction of the saccharine matter of the milk, as Beusch supposed to occur. Neither was the opinion confirmed by the experiments of Drs. Beusch, Playfair, and others that the fatty constituents of the milk are augmented by a vegetable, and diminished by an animal diet. By a change from an animal to a vegetable diet the quantity of the solid ingredients of the milk, namely, the fat and casein, was diminished, while the saccharine matter was somewhat increased. By fatty food the solid ingredients of the milk were but relatively increased, especially the butyraceous, while at the same time there was a decrease in the sugar.

3. The fact developed by the experiments of Dr. S., namely, that by animal food the quantity of butter in the milk is so much increased, would seem to prove that the fatty matter of the milk is formed, in a great measure at least, from the albumen.— *Vierteljahrschrift, f. d. Prakt. Heilk.*

On the 23rd of August, a statue to Laennec was inaugurated at Quimper, (Brittany), his native place, with imposing ceremonies. Orations were delivered on the part of the General Association of Physicians of France, of the Academy of Medicine, and of the Faculty of Medicine of Paris, of the profession at large, and of that of Brittany in particular, by Messrs. Tardieu, Roger, Bouillaud, Lediberder and Halleguen, respectively, followed by a grand banquet.

The discussion upon tuberculosis, which has occupied the attention of the Academy of Medicine (Paris) for several months past, and has elicited about all that is known about the matter, and more too, has at last been closed, but is likely to be re-awakened by the recent treatise of M. Villemin, in which he attempts to demonstrate the virulence and specificity of tuberculosis by establishing its analogy to glanders and syphilis, by means of inoculations; his conclusions, however, are disputed, and need confirmation.

M. Sichel (Paris) reports a case of encephaloid and melanotic tumor of the left orbit having crowded back and compressed the ocular globe. Extirpated by himself at his clinic, in 1859, resulting in apparent cure, followed by relapse after nine months, and a second extripation, and cauterization with chloride of zinc paste; the destruction by cancerous disease of the upper and internal portion of the orbital wall, which had not been subjected to the action of the zinc; the cerebral pulsations being visible through the encephaloid mass. The disease was again developed in its fullest intensity after two years (in 1861) and after a fall, from a wagon, upon the head, and again removed successfully in 1862. The learned professor reports the case as one of the most curious which has ever occurred to him in the course of his long and extensive practice.

Dr. Gent (Paris) reports the successful treatment of chlorosis by means of compressed air, attributing the success of this treatment to the increase in the respiratory capacity, by reason of the increased quantity of air introduced into the lungs, and to the augmented hæmatosis, due to more perfect oxydation.

M. A. Desprès has observed at the Hospital "Lourcine," Paris, seven cases of phagedenic chancres of the rectum consecutive upon soft chancres or ulcerated mucous patches of the anus. He has moreover seen eight cases of soft chancres, and two cases of mucous patches of the anus converted into phagedenic chancres.

He describes in a memoir (*Archiv. de Med.*, March 1868) every stage of the disease from the ulceration rapidly arrested by strong cauterization, to ulcerative, and subsequently fibrous, strictures of the rectum.

The caustic preferred by M. Desprès is a saturated solution of chloride of zinc, introduced into the anus by means of a pledget of lint, etc., etc. The author moreover states that if the ulceration be extensive, stricture is inevitable, and that there is no means of radically curing stricture of the rectum.

M. Scoutetten, in a paper read before the Academy of Sciences (Paris), Aug. 3rd, contests the propriety of the application of the term *Electrolysis*, and demands the substitution therefor of *Electrical absorption*, to the operative process applied to the removal of tumors, by means of the continuous electrical current. He claims with Faraday that the term *Electrolysis* signifies decomposition by electrical agency, as *analysis* that by chemical agency — and demonstrates the impossibility of effecting a true decomposition or *Electrolysis*, under the alleged conditions, and asserts that the removal of the morbid product is due to true physiological absorption, the result of the stimulating influences of the current upon the enfeebled vitality of the vessels of the part.

M. Personne, pharmaceutist to the Hospital la Pitié, has presented to the Academy of Medicine (Paris) a treatise on the impurities of commercial Chloroform. Chloroform containing water, he asserts, will, when exposed to the solar rays, become acid and evolve very irritating white vapors. M. Marsche has recognized among the products of this decomposition, alcohol, chlorohydric ether, chlorohydric acid and especially a large quantity of chloroxy-carbonic acid gas. M. Personne has verified these observations, but according to him they do not result from the decomposition of pure chloroform; they are due to the presence of a foreign substance, chloroxy-carbonic ether, accidentally present. In order to present this alteration it is necessary to agitate with the chloroform an alkali stronger than the alkaline carbonates, for example, caustic soda or potassa.

---

### PUBLISHER'S NOTICE.

We have at this office back numbers and volumes of the JOURNAL as follows:

Volume XVI (1859), all except the August number; Volume XVIII (1861), complete; Volume XIX (1862), February, March, June and July numbers; Volume XX (1863), complete; Volume XXI (1864), April, March, August, September, October, November and December numbers; Volume XXII (1865), all except July and August numbers; Volume XXIII (1866) complete; Volume XXIV (1867), complete; Volume XXV (1868), all except the number for January 15th.

Any number of any back volume, as above, will be mailed to any address on receipt of twenty cents. Any number from the present volume, with the exception of that for Jan. 15th, will be sent on receipt of fifteen cents.

Twenty-five cents will be allowed on account for each number of January 15th, 1868, received at this office.

